# The impact of the COVID-19 pandemic on physical activity, life satisfaction, anxiety, stress perception and coping strategies in student-athletes: A comparison between Belarus and Poland–countries with a different approach of anti-pandemic measures

**DOI:** 10.3389/fpubh.2022.1052744

**Published:** 2022-12-02

**Authors:** Andrei Shpakou, Dorota Sokołowska, Elżbieta Krajewska-Kułak, Mateusz Cybulski, Beata Kowalewska, Filip Korpak, Sergei Surkov, Jakub Owoc, Jan Krakowiak, Krystyna Kowalczuk

**Affiliations:** ^1^Department of Integrated Medical Care, Medical University of Bialystok, Białystok, Poland; ^2^Department of Physical Education and Tourism, University of Finance and Management, Bialystok, Poland; ^3^Department of Recreation and Tourism, Faculty of Physical Education and Health in Biala Podlaska, Jozef Pilsudski University of Physical Education in Warsaw, Warsaw, Poland; ^4^Department of Athletics, Swimming and Skiing, Faculty of Physical Education and Sports, Brest State A.S. Pushkin University, Brest, Belarus; ^5^Department of Gerontology, Public Health and Education, National Institute of Geriatrics, Rheumatology and Rehabilitation, Warsaw, Poland; ^6^Department of Social Medicine, Medical University of Lodz, Lodz, Poland

**Keywords:** students, physical activity, life satisfaction, stress, coping strategies, COVID-19, Poland, Belarus

## Abstract

**Background:**

More than two years of the COVID-19 pandemic has changed lives of people around the world and had a profound impact on the field of sports. This has resulted in decreased physical activity (PA) and changes in mental health. The goal was to assess self-reported physical activity, life satisfaction, perceived stress, choice of coping strategies and their correlations among student athletes from two neighboring countries facing different anti-pandemic strategies.

**Methods:**

Cross-sectional surveys using standardized questionnaires: International Physical Activity Questionnaire—Short Form (IPAQ-SF), Satisfaction With Life Scale (SWLS), State-Trait Anxiety Inventory (STAI), Perceived Stress Scale (PSS-10), and Coping Orientation to Problems Experienced (Mini-COPE) to compare 600 students from Physical Education and Sports departments of universities in Belarus (*n* = 333), where restrictions were found to be less stringent than in neighboring Poland (*n* = 267).

**Results:**

Minor differences in physical activities between both countries indicate that student athletes have adapted fairly quickly and found ways to keep their PA at a fairly high level. Nevertheless, higher PA was reported in the group of student athletes from Belarus. PA levels correlated with life satisfaction, anxiety and stress levels. Female students from Poland reported lower satisfaction with their lives. Their perception of stress was twice as high as that of their Belarusian counterparts. The most common coping strategy in both groups was active coping. Polish respondents less frequently used strategies of avoiding problems and seeking outside support.

**Conclusion:**

The level of physical activity and well-being of student athletes are associated with increased mental health and coping with stress. They also contribute to prevention of affective disorders during the COVID-19 pandemic. Moreover, it is dependent on the country's anti-pandemic policies.

## Introduction

The COVID-19 pandemic may be viewed as a universal stressor that threatens life and health at a population level (1, 2). Studies report not only socio-economic and health implications but also increasingly often a serious psychological impact on people (3, 4). Some of the factors associated with mental health problems include age, gender, education, close contact with COVID-19 infected individuals, exposure to news and social media, coping styles, as well as confidence in recommended personal protective measures (5). The COVID-19 pandemic has the most impact on the quality of social contacts, work or studies, physical activity and mental health score (1).

University students, especially physical education and sports students, are a group to most likely experience major changes in their daily lives in crisis situations (6, 7). During the COVID-19 pandemic they were exposed to additional stressors such as reduced physical activity due to interruptions in their training programs or isolation from team member and sports community (8, 9). Although most of such difficulties are common to athletes at any age or fitness level, previous studies have focused mainly on professional athletes.

Despite numerous studies on stress, distress or coping strategies during COVID-19, it is still not clear which factors may cause deterioration (10). The pandemic preventive measures were often taken on an international level affecting many countries and more than 1 billion students and schoolchildren (72.4%) (11). However, their extent varied between countries as the spread of SARS-CoV-2 virus was not the same everywhere in terms of time and scale, nor were capacity and readiness of health systems (12). The application of more or less restrictive pandemic containment strategies has had mixed effects on mental well-being of populations and public health in general. It is academically and practically interesting to compare the impact of a pandemic on mental health in countries with different COVID-19 prevention policies.

Belarus (BY) in Eastern Europe is one of the few countries that did not impose any lockdowns. Belarus has not endorsed quarantine and has proceeded with “life as usual” approach without closing borders, businesses, restaurants, museums, cinemas, schools or universities. Athletes were training, sporting competitions continued. Since the social distancing measures were not strictly implemented or enforced, it remained up to individuals whether and how to change their behavior patterns. This was a very liberal approach in comparison to neighboring Poland and many other countries.

During the same time Poland (PL) was in a lockdown. Universities and schools switched to remote learning, shopping malls and restaurants were closed, limits were placed on people in stores, churches and public transportation. Moreover, gatherings and mass events or even family celebrations were banned while state borders outside the Schengen Area were closed (13). As an overall result, countries with strict governmental restrictions and quarantine measures, high availability of medical services and effective state programs for the vaccination of citizens, were generally doing better in terms of the number of infected per capita (14).

This provided opportunity for various comparative studies investigating populations of closely situated cities in neighboring countries with different methods of pandemic control (15). This study compares impact of the pandemic and various restrictive measures implemented during the 2022 Omicron variant pandemic on the populations of two culturally neighboring regions: Western Belarus and Eastern Poland. This was accomplished through a joint cross-border effort using standardized methods (16) in two bordering regions of both countries.

The impact of anti-pandemic measures on young people who are developing dual careers (student and athlete) remains unclear, however this group is considered vulnerable to high levels of stress (17). It is therefore important to take into account their personal competence and provide support (18). Missed opportunities and uncertainty about future is sports may have negative impact on both, athletes themselves and a sporting industry (19). This combined with additional academic stress, social distancing, temporary restrictions on travel, fear of COVID-19, lifestyle changes, lack of physical activity, remote learning and complicated interactions with teachers and coaches significantly increased stress levels, enhanced anxiety and affected psychological health. In addition, it is worth noting that the official Belarusian media are more cautious in covering the pandemic and tend to downplay its impact and consequences compared to Poland (20, 21).

The study on mental health of Belarusian and Polish student-athletes may contribute to a better understanding of the relationship between the COVID-19 outbreak and public health problems since preventive measures implemented by Belarus (22) were very different from those used in most countries. We hypothesized that student-athletes would be more likely to seek to maintain pre-pandemic training conditions during the COVID-19 lockdown and perhaps this, despite limitations in general, would minimize adverse effects. It is important to monitor health status of young people in the era of the ongoing COVID-19 pandemic. So far, there were no studies that would compare student athlete populations of Eastern Poland and Western Belarus. This is to our best knowledge the first study on young athletes from both sides of EU eastern border.

The main objective of the study was to investigate self-reported rates of physical activity, life satisfaction, levels of anxiety and stress perception, as well as decisions on coping strategies and in student-athletes during the COVID-19 pandemic in two neighboring countries (Belarus and Poland) in the context of various anti-pandemic national policies. Other aims included identifying and investigating behavioral criteria and predictors that contribute to minimizing the adverse effects of the situation on mental health.

## Methods

### Survey design and participant recruitment

This study is based on a cross-sectional survey conducted as part of an international multicenter research project “The COVID-19 Coping Study of Students from East Europe (SEECoping-S).” The web study was conducted in January-March 2022, a time when the Omicron virus species was prevalent and there was a significant increase in the incidence of the disease. Data source location: the sociological survey was conducted among students of partner universities on both sides of the eastern border of the European Union, in the border cities of Belarus and Poland. Region: Europe, Country: West Belarus and East Poland (universities with faculties of physical culture and sport in Grodno, Brest, Elblag, Bialystok and Biala Podlaska). The institutions were chosen, as despite the universities being located a relatively small distance from each other, they are in different countries and subject to different anti-pandemic strategies. An invitation to participate in an online survey (Google forms were used as a survey platform for electronic distribution) was distributed through targeted advertising including an e-learning platform (Moodle), Skype, Microsoft Teams, and university social networks.

Prior to the start of the study, all participants were informed of its objectives, methodology, and the anonymous and confidential nature of the survey. All participants provided informed consent. The questionnaire contained information about the study, its goals, objectives, an invitation to participate, as well as socio-demographic data on age, gender, education profile, self-reported physical activity, contact with individuals with COVID-19, being in self-isolation, being in quarantine, infection with the SARS-CoV-2 virus, disease severity, and vaccination course. The survey with sociodemographic questions was previously developed and reviewed by experts in the area of physical activity, sport exercise and psychology. The consequences of the COVID-19 pandemic and its prevention measures were seen as major stressors affecting the daily life of the target group.

The main part of the questionnaire included a set of generally accepted standardized questionnaires in the Russian, Belarusian and Polish versions in order to determine the level of declared physical activity, life satisfaction, state and trait anxiety, assessment of stress experience, and the use of coping strategies. Russian and Belarusian are the state languages in Belarus. If necessary, Russian and Belarussian language equivalents of the Polish language statements were created by employing a back-translation method (23), which required a speaker fluent in both Polish and Russian and Belarusian to translate the Polish version of the questionnaire into a Russian and Belarusian version. Subsequently, a second bilingual individual translated the Russian and Belarusian versions of the questionnaire back into Polish.

Permission was obtained from the leadership of the universities participating in the study to conduct an anonymous survey of students. The research was undertaken in accordance with the Declaration of Helsinki and its subsequent amendments. Participation was entirely voluntary, anonymous, and consensual; informed consent from participants was obtained prior to testing. No financial incentives were offered or provided for participation. The survey did not collect any identifiable information from the participants. Ethical permission to conduct the study was obtained from the Bioethical Review Board at the Medical University of Bialystok, Poland (document number: APK. 002. 1932. 2022).

### Study questionnaire

Personal Information Form: The personal information form, which was created by the researchers in line with the literature, consisted of 5 questions about the sociodemographic characteristics of the individuals, 6 questions about COVID-19 pandemic, and four groups of questions from standardized questionnaires on physical activity and mental health measures.

The International Physical Activity Questionnaire- Short Form (IPAQ-SF) was used to assess Physical Activity (PA). This questionnaire is composed of questions related to specific types of PA, e.g., moderate, higher activities, and walking, in terms of the frequency and duration of each specific type of activity, and the time spent seated per day in a week (24). Total energy expenditure herein is calculated by multiplying the frequency and duration of PA with the corresponding intensity expressed in metabolic equivalent of task units (METs), and then summing the results for all activities performed during the week. Levels of PA were classified as high (intense, vigorous PA), sufficient (increased and moderate PA), and low (insufficient PA) (25). Cronbach's (α) reliability analysis was applied in order to verify the internal consistency of the questionnaire: the reliability of the tool was assessed as α = 0.756, which is a satisfactory level of reliability.

The Satisfaction With Life Scale (SWLS) proposed by E. Diener et al. (26) was employed to assess cognitive judgments about the subjective perception of well-being in life (27, 28). Life Satisfaction was measured using the languages versions of the SWLS, comprised of 5 items rated in a 7-point Likert scale ranging from 1 (strongly disagree) to 7 (strongly agree, greater satisfaction). The resulting measurement is an overall index of satisfaction with life. Depending on the degree of satisfaction with life, the results were classified as low, medium and high. For the purpose of further analysis, we dichotomized the SWLS scores into Lower (SWLS < 19), Intermediate (SWLS = 20–25) and Higher (SWLS > 25) (29). In our study, the Cronbach alpha Coefficient for STAI was 0.859.

The State-Trait Anxiety Inventory (STAI) (30) was implemented for investigating anxiety understood as a temporary and situation-related state of an individual, and anxiety as a relatively stable personality trait. The STAI was developed by Spielberger and adapted to Polish conditions (31), while the Russian version was adapted by Khanin (32). To assess the intensity of actual anxiety, the first part of STAI (Form X_1_) was utilized, while Form X_2_ was employed to assess dispositional or general anxiety (30). Each subscale of the questionnaire consists of 20 items that take the form of short statements relating to an individual's subjective feelings. For each of them, the subject is supposed to select one of four categorized answers. Anxiety levels up to 30 points are considered low, 30 to 45 are considered moderate, and 46 and above are considered high. The minimum score on each scale is 20 points, with a maximum of 80 points. Similar to the original tool, the Polish and Russians versions of STAI are characterized by satisfactory psychometric properties. In this study, the Cronbach alpha Coefficient for STAI was 0.95.

The perceived stress level was evaluated by the Perceived Stress Scale (PSS-10) (33, 34). PSS-10 has the most satisfactory psychometric properties for measuring stress. It consists of six positively worded items and four negatively worded items. Each item in the PSS-10 is rated on a 5-point Likert scale ranging from never (0 points) to very often (4 points). A two-factor PSS-10 model (perceived helplessness as a positive factor and perceived self-efficacy as a negative factor) has been identified (35). The degree of subjective perception of life was determined in 5 gradations, ranging from prosperous, to overloaded with stress. Initially, the results of PSS-10 were assessed *via* subscales: “Overload,” which measures the subjectively perceived level of tension in the situation, and ”Stress response,” which determines the level of efforts made to overcome stress. The overall result characterized the degree of perceived stress in a gradation from the minimum, to the maximum. In this study, the Cronbach alpha Coefficient for PSS was 0.781.

Coping Orientation to Problems Experienced (Mini-COPE). The tool is used to assess typical ways of responding when experiencing severe stress. The multidimensional self-report coping inventory (Mini-COPE) by Carver (36) was adapted by Juczyński, Ogińska-Bulik (37) and Rasskazova (38). When completing the test, the respondent should indicate how often each strategy is used in a very difficult situation. In our study, 14 coping strategies was assessed using the shortened version recommended in 1997 (36). Here, the higher the score, the more often the subject uses the strategy.

Each strategy is evaluated separately based on the average number of points obtained from the two statements assigned to it. The higher the score, the more often the individual uses this strategy to resolve a stressful situation. The severity of the scales of coping behavior was classified as: 1. Active coping (actions to eliminate, reduce the stressor or its consequences). 2. Planning (thinking about and planning what to do). 3. Positive reframing (thinking about a negative or challenging situation in a more positive way). 4. Acceptance (accepting the situation as irreversible, which one needs to get used to it). 5. Humor (as a way to soften unwanted emotions). 6. Religion (as a source of emotional support and a pointer to a positive reappraisal). 7. Use of emotional support (sympathy, understanding, moral support). 8. Use of instrumental support (the desire to get advice, help or reliable information). 9. Self-distraction (avoiding thoughts about the situation by engaging in other activities). 10. Denial (denial of the reality of a stressful situation, ignoring it). 11. Venting (focus on emotions and their manifestation, worrying about one's emotions, a tendency to discharge them). 12. Substance use (use of alcohol or other psychoactive drugs). 13. Behavioral disengagement (“helplessness,” ”submission,” “refusal of efforts”). 14. Self-blame. All responses were grouped into four integral coping strategies: Active coping, Helplessness, Seeking support, Avoidance coping (37). In this study, the Cronbach alpha Coefficient for Mini-COPE was 0.830.

### Statistical analysis

Statistical analyses were carried out using the STATISTICA software package ver. 13.0. All analyses were adjusted for gender and countries, as these were considered a priori to be potential confounders. The Shapiro-Wilk test was applied to check normality. Distribution of the quantitative data appeared to diverge from the normal pattern. Therefore, methods of non-parametric and parametric statistics were used. Quantitative variables were presented considering descriptive characteristics: mean (M), standard deviation (SD), and median (Me). Comparative analysis between the BY and PL groups was performed using the *T*-test for independent samples. In cases of large SD values, the non-parametric Mann-Whitney U test was additionally applied, while frequencies and percentages were used for qualitative variables. Differences in categorical variables were assessed using the Pearson's χ2 (Chi-square) tests. Correlations between qualitative variables were calculated using the rho-Spearman coefficient, which measures the strength and direction of correlations between variables. The interval estimate of the statistical parameters was determined using the 95% confidence interval. For all analyses, values of p < 0.05 were considered statistically significant.

## Results

### Demographics

The survey was completed by 600 students. The Belarusian group consisted of 333 students of physical education aged 18 to 35 (M = 20.9; SD = 3.71), including 167 men (50.2%) and 166 women (49.8%) who practiced recreational sports (amateurs). The Polish group consisted of 267 respondents aged 18 to 36 (M = 21.9; SD = 3.82), including 123 women (46.1%) and 144 men (53.9%). Participants from Belarus were younger than Polish. The proportion of men and women in both groups did not differ significantly (χ2 = 0.8; *p* = 0.4). Self-isolation and quarantine were cited by respondents as the most effective ways to prevent COVID-19. Belarusian female student athletes were more likely to resort to self-isolation, which may be due to the more frequent use of laboratory diagnostics during competition. Sports competitions were not banned in Belarus. As for quarantine, female athletes from Poland reported more frequent use of it. The low vaccination rate (about 50% in both groups) is noteworthy. No differences were found by sex or country.

All descriptive and statistical results are described in Table 1.

**Table 1:** Cohort demographics, COVID-19 exposure and differences between Belarus and Poland.

**Variables (*n*, %, 95%CI)**	**Belarus**, ***N** =* **333 (55.5%)** **(BY)**		**Poland**, ***N** =* **267 (44.5%)** **(PL)**		**Total sample** ***N** =* **600** **(100%)**		**χ2 for country comparison** ** (BY vs. PL)**
	**Male** **(*****N** **=*** **167)**	**Female** **(*****N** **=*** **166)**	**Male** **(*****N** **=*** **144)**	**Female** **(*****N** **=*** **123)**	**Male** **(*****N** **=*** **311)**	**Female** **(*****N** **=*** **289)**	
Age, mean (years ± SD)	21.1 ± 3.53	20.8 ± 3.89	22.1 ± 3.77	21,4 ± 3.84	21,0 ± 3.87	21,7 ± 3.69	
	20,9 ± 3.71		21,9 ± 3.82		21.4 ± 3.79^+^		Mann–Whitney U test: U = 34139.5, P < 0.001
Diagnosed with COVID 19^α^ (infection with SARS-CoV-2)	29 17.4; (11.6–23.1)	44; 26.5; (19.8–33.2)	18; 12.5; (7.1–17.9)	32; 26.0; (18.3–33.8)^*^	47; 15.1; (11.1–19.1)	76; 26.3; (21.2–31.45)^*^	^*^χ2 = 6.3; P < 0.05
	73; 21.9; (17.5–26.4)		50; 18.7; (14.1–23.4)		123; 20.5; (17.3–23.7)^^+^^		χ2 = 119.0; P < 0.001
Quarantine	91; 54.5; (46.9–62.0)	100; 60.2; (52.8–67.7)	49; 34.0; (26.3–41.8)	65; 52.8; (44.0–61.7)^*^	140; 45.0; (39.5–50.6)	165; 57.1; (51.4–62.8)^*^	χ2 = 7,6; P < 0.01
	191; 57.4; (52.1–62.7)		114; 42.7; (36.8–48.6)		305; 50.8; (46.8–54.8)^^+^^		χ2 = 5.2; P < 0.05
Vaccinated against COVID−19	90; 53.9; (46.3–61.5)	89; 53.6; (46.0–61.2)	63; 43.8; (35.7–51.9)	61; 49.6; (40.8–58.4)	153; 49.2; (43.6–57.8)	150; 51.9; (46.1–57.7)	P > 0.05
	179; 53.8; (48.4–59.1)		124; 46.4; (40.5–52.4)		303; 50.5; (46.5–54.5)		P > 0.05

N is the number of observations; % is the percentage of the total number of study participants in a given group.

95%CI0, 95-percent confidence interval; SD, standard deviation.

α , The study population consisted of respondents with SARS-CoV-2 confirmed by reverse transcription polymerase chain reaction (RT-PCR) tests.

* Differences in relation to gender are significant.

+ differences between Polish students and students from Belarus are significant (p < 0.05).

The differences in frequency of individual symptoms suggest that despite the relatively high percentage of COVID-19 diagnoses, pathognomonic symptoms and their combination (decreased sense of taste, fever, sore throat, fatigue, headache, shortness of breath, wheezing and cough) were less pronounced in student athletes from both study groups.

### Physical activity

The results of the analysis of the weekly time spent on PA indicate that daily intense physical activity, which is accompanied by rapid breathing, accelerated heartbeat and sweating, was not declared by the respondents only in 9.8% of all cases (BY = 8.7%, PL = 11.2%). Moderate PA, which requires medium effort, accompanied by some increase in breathing and not excessive sweating (for example, carrying light objects, cycling at a normal pace, or practicing in amateur sports sections), was stated as practiced by 93.4% of the respondents (BY = 95.2%, PL = 91.1%). The third type of analyzed activity was walking at home, during recreation and exercise. This activity was noted to have been followed by almost all respondents (99.8%). During COVID-19 quarantine and isolation, there was a significant decrease of moderate-intensity physical activity.

**Figure 1:**
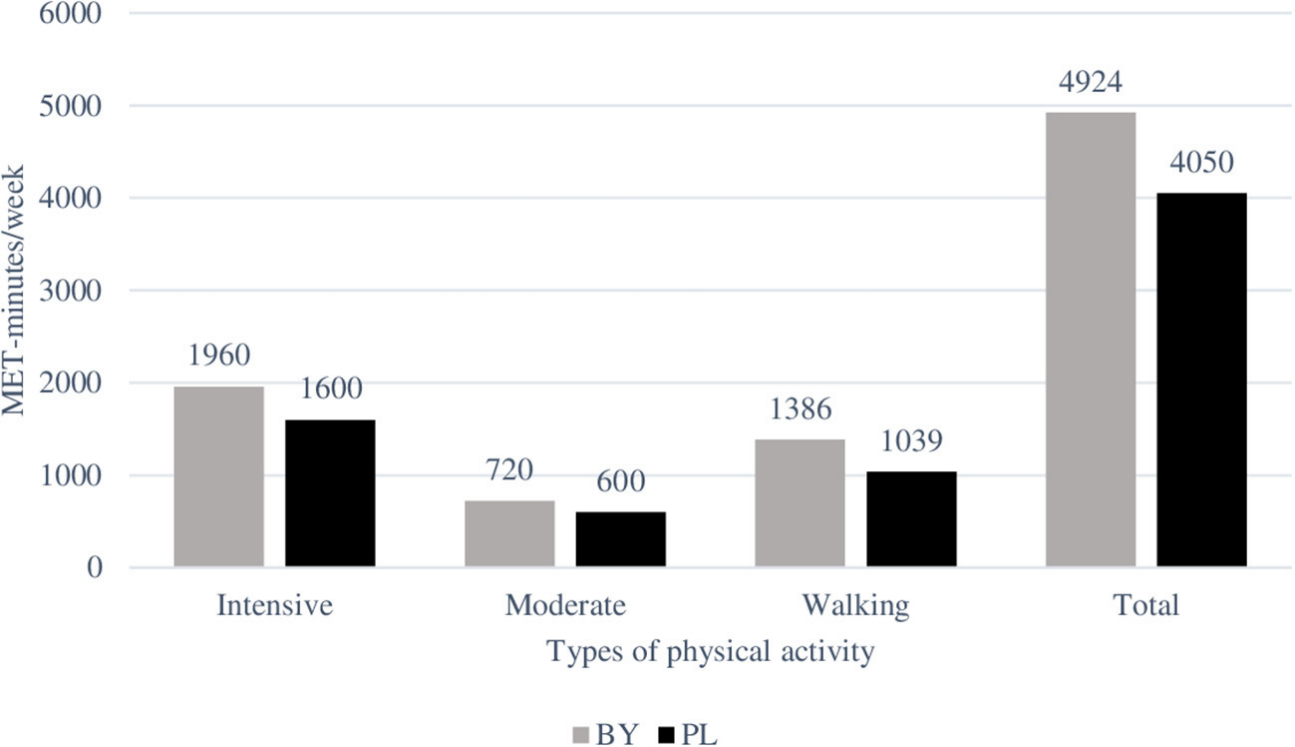
Proportion of different types of physical activity PA declared by respondents.

Figure 1 shows the distribution of PA levels in BY and PL groups of students in medians. In terms of intensity of physical activity, the majority of respondents met the IPAQ criteria (31) for a sufficient and high level. The total PA index was 5764.3 ± 4822.0 MET-minutes/week. Among students from PL, it turned out to be lower, at 5065.5 ± 4224.4, among students from BY—higher, at 6324.6 ± 5191.0 MET-minutes/week. Despite various restrictions imposed during the COVID-19 pandemic, respondents from both countries tended to adhere to high and moderate levels of physical activity, as evidenced by MET-minutes/week (Table 2).

**Table 2:** Descriptive statistics of the main types of PA of Belarusian and Polish students-athletes, MET-minutes/week (M ± SD).

**Physical activity**	**Group, countries**	**Gender**	**M ±SD**	**P-test probability value calculated using** ***t*****-test**	
Intensive	BY	Male (1)	3007.8 ± 2832.5	P_1[BY,PL]_ < 0.05 P_2[BY,PL]_ > 0.05 P_[BY,PL]_ < 0.05	P_[1.2]_ < 0.05
		Female (2)	2444.1 ± 3003.1		
		Total	2676.6 ± 2923.0		
	PL	Male (1)	2762.8 ± 2838.0		P_[1.2]_ < 0.001
		Female (2)	1951.0 ± 2766.2		
		Total	2388.8 ± 2821.1		
	Total	Male (1)	2840.6 ± 2831.4		P_[1.2]_ < 0.001
		Female (2)	2234.3 ± 2910.1		
Moderate	BY	Male (1)	1290.1 ± 1648.8	P_1[BY,PL]>_ 0.1	P_[1.2]_ > 0.05
		Female (2)	1883.3 ± 1732.7	P_2[BY,PL]_ < 0.05	
		Total	1236.8 ± 1689.5	P_[BY,PL]_ < 0.05	
	PL	Male (1)	1190.2 ± 1640.2		P_[1.2]_ < 0.01
		Female (2)	714.4 ± 919.2		
		Total	971.0 ± 1374.9		
	Total	Male (1)	1243.8 ± 1642.9		P_[1.2]_ < 0.001
		Female (2)	983.7 ± 1460.1		
Walking	BY	Male (1)	2441.3 ± 3064.0	P_1[BY,PL]_ < 0.05	P_[1.2]_ > 0.05
		Female (2)	2411.1 ± 2923.8	P_2[BY,PL]_ < 0.05	
		Total	2676.6 ± 2923.0	P_[BY,PL]_ < 0.01	
	PL	Male (1)	1680.0 ± 2241.7		P_[1.2]_ > 0.05
		Female (2)	1735.7 ± 2144.2		
		Total	1705.07 ± 2193.4		
	Total	Male (1)	2088.8 ± 2736.8		P_[1.2]_ > 0.05
		Female (2)	2106.3 ± 2547.9		
Total physical activity	BY	Male (1)	6639.1 ± 5591.3	P_1[BY,PL]_ < 0.05	P_[1.2]_ > 0.05
		Female (2)	6008.2 ± 24750.4	P_2[BY,PL]_ < 0.01	
		Total	6324.6 ± 5191.0	P_[BY,PL]_ < 0.01	
	PL	Male (1)	5639.2 ± 4413.9		P_[1.2]_ < 0.05
		Female (2)	4401.2 ± 3904.7		
		Total	5065.5 ± 4224.4		
	Total	Male (1)	6173.2 ± 5097.1		P_[1.2]_ < 0.01
		Female (2)	5324.2 ± 4474.5		

Maintaining regular physical activity is an important preventive strategy for physical and mental health during anti-pandemic restrictions. None of the respondents in the student athlete groups showed insufficient physical activity. Most respondents declared sufficient and elevated PA levels. Among women, it was characteristic that Polish female students included twice as many persons with sufficient activity, but fewer with high activity. A more rational position was found in men. They were quicker to optimize their PA levels. The percentage of high PA turned out to be similar in both cases (73.9% among Belarusian students vs. 68.9% in Polish students. The situation was similar for moderate activity (about 15% in both groups) and low activity (11.4 vs. 16.1%). The small differences in PA between respondents from the two countries indicate that student athletes adapted fairly quickly and found ways to quickly improve their PA levels, such as by training at home.

### Life satisfaction scale (SWLS)

The study found that the majority of responding university student-athletes were mostly satisfied with life (68.5%). Statistical scores obtained from the total SWLS mean score was 25.0 ± 9.97. However, student-athletes from Belarus were most satisfied with life (26.0 ± 5.93 vs. 23.8 ± 5.81) (*p* < 0.001).

Extreme dissatisfaction with life (5–9 points) was found only in 1.0–1.4% of cases. A high degree of dissatisfaction (10–14 points) was observed in 2.0% of all cases in Belarus and 5.6% of all cases in Poland, while an average degree of dissatisfaction (15–19 points) was in 8.7 and 12.0%, respectively. An indifferent answer (20 points), indicating that the respondent does not experience either dissatisfaction with life or satisfaction was reported among 17.1 vs. 21.6%. Moderate (21–25 points) and high (26–30) satisfaction were noted in 24.0 vs. 28.9% and 21.6 vs. 21.7% of all observations, respectively. More 30 points-−22.2 vs. 10.5%. The classical division into Lower (SWLS < 19), Intermediate (SWLS = 20–25), and Higher (SWLS > 25) is shown in Table 3.

**Table 3:** SWLS scores for study groups by gender and country (N, %, 95%CI).

**Groups of students-athletes**	**Gender**	**SWLS (points)** ** M ±SD**	**Lower** ** (SWLS < 19)**	**Intermediate Scores (SWLS = 20–25)**	**Higher (SWLS > 25)**
Belarussians students [BY]	Male (*N =* 167)	26.4 ± 6.17	23; 13.8 (8.5–19.0)	49; 29.3 (22.4–36.3)	95 56.9 (49.4–64.4)
	Female (*N =* 166)	25.6 ± 5,66	24; 14.5 (9.1–19.8)	52; 31.3 (24.3–38.4)	90; 54.2 (46.6–61.8)
	Total	26.0 ± 6.95	47; 14.1 (10.4–17.9)	101; 30.3 (25.4–35.3)	185; 55.6 (50.2–60.9)
Polish students [PL]	Male (*N =* 144)	24.2 ± 5.70	27; 18.8 (12.4–25.1)	57; 39.6 (31.6–47.6)	60; 41.7 (33.6–49.7)
	Female (*N =* 123)	23.3 ± 5.93	30; 24.4 (16.8–32.0)	48; 39.0 (30.4–47.6)	45; 36.6 (28.1–45.1)
	Total	23.8 ± 5.81	57; 21.3 (16.4–26.3)	105; 39.3 (33.5–45.2)	105; 39.3 (33.5–45.2)
Total	Male (*N =* 311)	25.3 ± 6.05	50; 16.1 (12.0–20.2)	106; 34.1 (28.8–39.4)	155; 49.8 (44.3–55.4)
	Female (*N =* 289)	24.6 ± 5.88	54; 18.7 (14.2–23.2)	100; 34.6 (29.1–40.1)	135; 46.7 (41.0–52.5)
	Total	25.0 ± 5.97	104; 17.3 (14.3–20.4)	206; 34.3 (30.5–38.1)	290; 48.3 (44.3–52.3)
*T*-test and Chi^2^	P_1, BY − PL_ < 0.01; P_2, BY − PL_ < 0.01, P_BY − PL_ < 0.001		χ^2^ = 5.4; P_BY − PL_ < 0.05	χ^2^ = 5.3; P_BY − PL_ < 0.05	χ^2^ = 15.6; P_BY − PL_ < 0.001

The Belarusian group had the highest percentage of participants reporting satisfaction according to the SWLS (56.6%), with 39.3% of all respondents being the most satisfied with their lives (SWLS > 25).

The relationship between physical activity and life satisfaction was not clear in any of the student athlete groups. Respondents with high PA accounted for less than half (42.3%) of the students who were most satisfied with their lives. Those who contracted and survived COVID-19 reported a dramatic decrease in physical activity and life satisfaction. Vaccination, on the other hand, had a positive effect on both physical activity levels and life satisfaction. A fairly strong correlation between physical activity and life satisfaction was found only for women. The correlation was weak or very weak in all other observations.

### Anxiety

The specific effect of the anti-pandemic measures becoming more restrictive was significantly reflected in an important mental health indicator of anxiety (trait and state), among other issues. Statistical results obtained for overall mean trait anxiety were 38.84 ± 11.51, while that for state anxiety were 43.35 ± 10.29. The increase in state anxiety was greater than the increase in trait anxiety, which may indicate the trait's entrenchment and chronic nature of the process. Accordingly, the overall prevalence of high anxiety trait (> 46 points) among students was 29.2%, and the anxiety state was 42.0%. Student athletes from Poland had higher levels of COVID-19-related anxiety (trait and state) than did respondents from Belarus. When considering the normal values for both sexes, high levels of anxiety were found in both male and female groups. However, women were more likely to have severe or moderate anxiety symptoms. One-way analysis of variance showed that women were more likely than men to experience higher levels of anxiety related to the COVID-19 pandemic during these times.

The prevalence of anxiety (trait) expressed as a percentage was higher in women-−53.6% (*p* < 0.001) than in men-−31.2% (*p* < 0.001). The prevalence of anxiety (trait) as such was higher in women−32.5% (*p* < 0.01) than in men-−26.1% (*p* < 0.01). Similar results were obtained for anxiety (condition). Basic descriptive values and comparisons of the intensity of anxiety related to the COVID-19 pandemic by country and gender are presented in Table 4.

**Table 4:** Comparison of trait anxiety and state anxiety scores and country and gender of the respondents (M ± SD).

**Variation in state anxiety**	**BY**		**PL**		**Total sample**		**P**
	**Male** ** (*N* = 167) (1)**	**Female** ** (*N* = 166) (2)**	**Male** ** (*N* = 144) (1)**	**Female** ** (*N* = 123) (2)**	**Male** ** (*N* = 311) (1)**	**Female** ** (*N* = 289) (2)**	
Anxiety (trait)	36,51 ± 10.48	38,70 ± 11.21	37,85 ± 10.58	43,35 ± 13.05	37,13 ± 10.53	40.68 ± 12.23	P_PL[1.2]_ < 0.001
	37.60 ± 10.89		40,38 ± 12.08		38,84 ± 11.51		P_[1.2]_ < 0.001 P_2[BYPL]_ < 0.001 P_[BYPL]_ < 0.01
**Anxiety levels (trait) (** ***N*** **, %, 95%CI)**							
Low (< 30)	93; 27.9 (23.1–33.8)		54; 20.2; (15.4–25.0)		147; 24.5 (21.1–27.9)		P_[BYPL]_ < 0.05
Moderate (30–45)	151; 45.3 (40.0–50.7)		127; 47.6 (41.6–53.6)		278; 46.3 (42.3–50.3)		N/S
High (> 45)	89; 26.7 (22.0–31.5)		86; 32.2 (26.6–37.8)		175; 29.2 (25.5–32.8)		N/S
Anxiety (state)	39,56 ± 10.68	46,0 ± 9.44	41.13 ± 9.41	47,52 ± 10.76	40.29 ± 9.57	46.65 ± 10.04	P_BY[1.2]_ < 0.001 P_PL[1.2]_ < 0.001
	42.77 ± 10.08		44,07 ± 10.53		43.35 ± 10.29; 43; 14		P_[1.2]_ < 0.001
**Anxiety levels (state) (** ***N*** **, %, 95%CI)**							
Low (< 30)	42; 12.6 (9.0–16.2)		20; 7.5 (4.3–10.7)		62; 10.3 (7.9–12.8)		P_[BYPL]_ < 0.05
Moderate (30–45)	152; 45.6 (40.3–51.0)		134; 50.2 (44.2–56.2)		286; 47.7 (43.7–51.7)		N/S
High ( > 45)	139; 41.7 (36.4–47.0)		113; 42.3 (36.4–48.3)		252; 42.0 (38.1–46.0)		N/S

**P—**test probability value calculated using t-test and χ^2^ for country comparison.

Differences between the groups from both countries were related to anxiety levels, with students from Belarus reporting higher levels of physical activity showing lower anxiety levels (r = – 0.241, *p* < 0.05). A decrease in physical activity among Polish students was accompanied by an increase in both trait and state anxiety (r = −0.246, *p* < 0.05). The inverse correlation between life satisfaction and Spilberger test scores was even stronger (r = −0.458, *p* < 0.05).

### Perceived stress scale (PSS-10)

Statistical scores obtained from the total PSS-10 mean score was 18.9 ± 6.76. An increase in stress scores indicates an increase in stress intensity.

The majority of the students in both countries reported moderate perceived stress. Overall, 16.7 and 73.2% of the respondents reported low and moderate perceived stress, while 10.2% of all students experienced stress (PSS-10 < 27). Polish students' perception of stress was significantly higher than that of their Belarusian colleagues (14.6 vs. 6.6%). The minimal level was noted among Belarusian students (23.4 vs. 8.2%). The results of the “Overload” subscale assessment confirmed a higher level of stress among students from Poland. For the entire study group, the mean Perceived stress level score was 18.9 ± 6.76. The Belarusian students' scores were more favorable-−17.5 ± 7.07 vs. 20.6 ± 5.93 (*p* < 0.001). There were statistically significant differences between the genders. The scores of female students were also significantly higher than those of male students. The mean PSS-10 result was 17.7 ± 6.86 for men, and 20.2 ± 6.42 for women (*p* < 0.001).

The study attempted to assess correlations between the physical activity and the applied mental health indicators. Most correlations have insignificant relationship strength. There is a negative correlation at r = −0.109, *p* < 0.05 between PSS-10 test scores and physical activity. Students declaring high levels of PA were characterized by high resistance to stress (r = 0.123, *p* < 0.05). There was also a negative correlation between the level of PA and the severity of perceived stress (“Perceived stress” sub-scale–r = −0.122, *p* < 0.05, indicating that stress levels decreased as PA increased. A stronger positive correlation at r = 0.158 and r = 0.229 (*p* < 0.05) was observed between PSS-10 and STAI (trait and state) test scores. There was also a relatively close negative correlation at r = −0.296 between stress and life satisfaction. Accordingly, the higher the level of stress, especially the “Overload subscale,” the lower the level of life satisfaction (r = −0.429, *p* < 0.05). The “Stress response” subscale is also correlated with life satisfaction at r = 0.186, *p* < 0.05). This means that the higher the life satisfaction, the more frequently and intensively students use coping strategies aimed at proactive measures to prevent stress.

The adaptive coping of student athletes with acceptance and self-control directly correlates with maintaining optimal physical activity during the COVID-19 pandemic. To further clarify stress coping scenarios, it was necessary to rank the selected methods, which was achieved by assessing coping strategies using Coping Orientation to Problems Experienced (Mini-COPE).

### The choice of coping strategies

**Figure 2:**
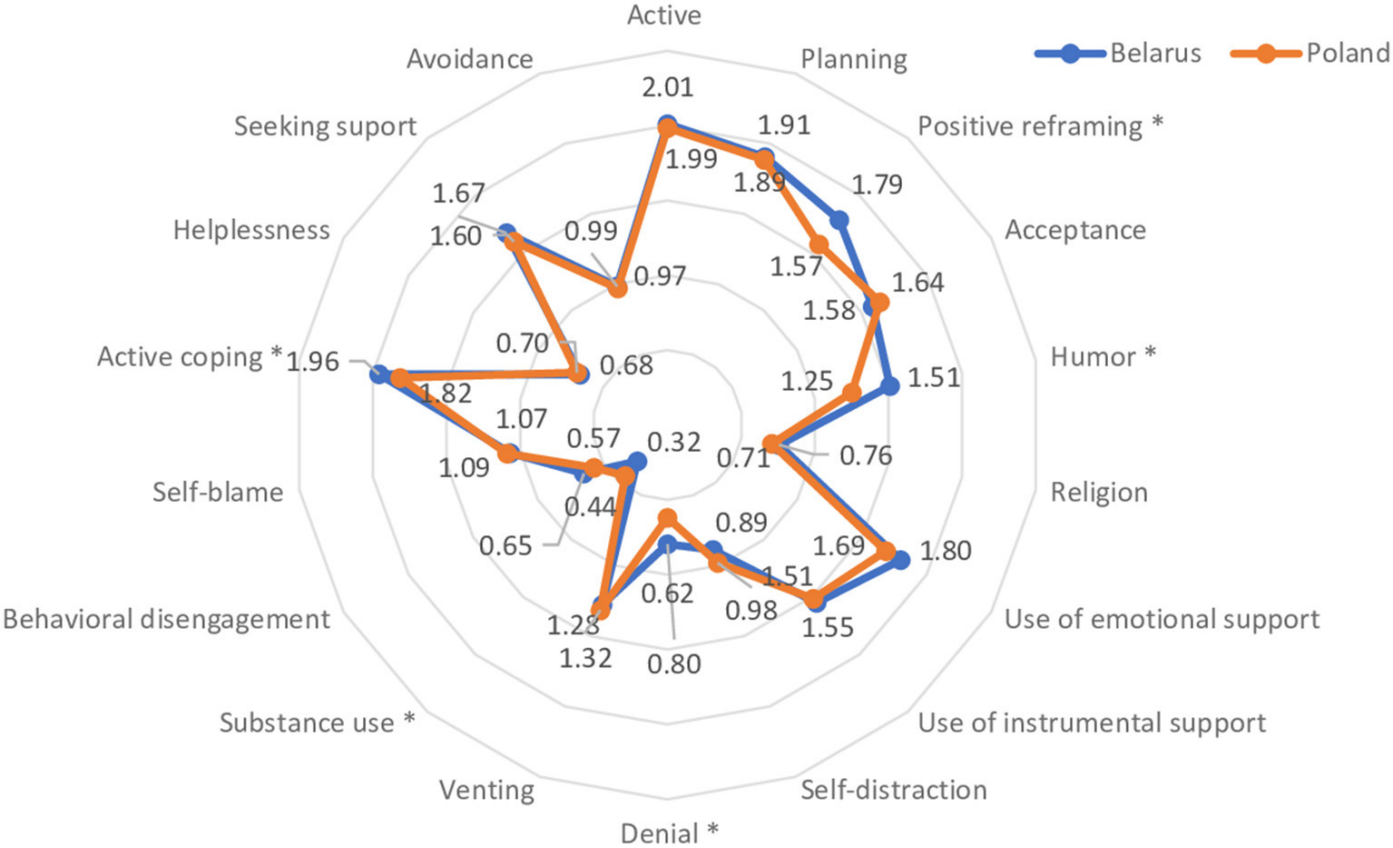
Scores for individual strategies for coping with stress (Mini-COPE). A higher value indicates a more frequently used behavior. The * symbol indicates the differences between the groups which are statistically significant.

A comparative analysis of stressful situation management by student-athletes in Belarus and Poland was conducted. The respondents indicated that they most often counter stress by active coping, i.e., taking action to improve the situation; planning, i.e., thinking about and planning what to do; positive reframing, and use of emotional support. Statistically significant differences between Belarusian and Polish respondents were observed on four scales: positive reframing, humor, emotional support and denial. For all these, the levels among Belarusian student-athletes were higher. In second place after active strategies, Belarusian respondents often denied the reality of a stressful situation by ignoring it. Figure 2 shows the distribution of the frequency of choice of coping strategies by students, taking into account the belonging to one of two groups according to country.

Table 5 shows the distribution of the frequency of choice of coping strategies among the male and female students surveyed, as well as significant differences in the two countries.

**Table 5:** Results of the rating choice of strategies for coping with perceived stress (with gender inclusion) (M ± SD).

**Coping-strategy**	**BY**		**PL**		**Total sample**		Significant differences in the groups^**^
	**Male (1)**	**Female (2)**	**Male (1)**	**Female (2)**	**Male (1)**	**Female (2)**	
1. Active coping	2.02 ± 0.80	2.01 ± 0.70	2.04 ± 0.73	1.92 ± 0.76	2.03 ± 0.77	1.97 ± 0.72	N/S
2. Planning	1.93 ± 0.80	1.88 ± 0.67	1.87 ± 0.80	1.19 ± 0.79	1.90 ± 0.80	1.89 ± 0.72	N/S
3. Positive reframing	1.73 ± 0.89	1.84 ± 0.78	1.53 ± 0.85	1.62 ± 0.81	1.64 ± 0.88	1.75 ± 0.80	P_BY1 − PL1_ < 0.001; P_BY2 − PL2_ < 0.001 P_BY − PL_ < 0.001
4. Acceptance	1.50 ± 0.84	1.67 ± 0.72	1.69 ± 0.82	1.58 ± 0.73	1.59 ± 0.81	1.63 ± 0.72	P_BY1 − PL1_ < 0.001
5. Humor	1.54 ± 0.93	1.48 ± 0.83	1.24 ± 0.91	1.27 ± 0.90	1.40 ± 0.93	1.39 ± 0.87	P_BY1 − PL1_ < 0.001 P_BY2 − PL2_ < 0.001 P_BY − PL_ < 0.001
6. Religion	0.83 ± 0.94	0.69 ± 0.86	0.69 ± 0.87	0.73 ± 0.86	0.76 ± 0.91	0.71 ± 0.86	N/S
7. Use of emotional support	1.72 ± 0.87	1.87 ± 0.74	1.54 ± 0.83	1.85 ± 0.80^*^	1.64 ± 0.85	1.87 ± 0.76^*^	P_BY − PL_ < 0.001
8. Use of instrumental support	1.51 ± 0.81	1.58 ± 0.68	1.38 ± 0.79	1.68 ± 0.82^*^	1.45 ± 0.80	1.62 ± 0.74	P_BY − PL_ < 0.001
9. Self-distraction	0.86 ± 0.66	0.92 ± 0.53	0.92 ± 0.62	1.05 ± 0.63	0.89 ± 0.64	0.97 ± 0.58	N/S
10. Denial	0.82 ± 0.71	0.77 ± 0.70	0.60 ± 0.68	0.63 ± 0.65	0.72 ± 0.70	0.71 ± 0.68	P_BY1 − PL1_ < 0.001 P_BY − PL_ < 0.001
11. Venting	1.19 ± 0.69	1.36 ± 0.61^*^	1.20 ± 0.72	1.45 ± 0.71^*^	1.19 ± 0.71	1.40 ± 0.65^*^	P_BY − PL_ < 0.001
12. Substance use	0.37 ± 0.70	0.26 ± 0.54	0.43 ± 0.67	0.46 ± 0.67	0.40 ± 0.69	0.34 ± 0.60	P_BY2 − PL2_ < 0.001
13. Behavioral disengagement	0.62 ± 0.7	0.67 ± 0.63	0.50 ± 0.62	0.65 ± 0.62^*^	0.56 ± 0.67	0.66 ± 0.63^*^	P_BY − PL_ < 0.001
14. Self-blame	1.03 ± 0.81	1.10 ± 0.80	1.04 ± 0.79	1.15 ± 0.91	1.04 ± 0.80	1.12 ± 0.85	N/S

M, arithmetic mean; SD, standard deviation.

* Differences between males and females within each country are significant (P < .05);

** P, test probability value calculated using Mann-Whitney test.

## Discussion

The more than two years of the COVID-19 pandemic has changed the lives of people around the world and has had a profound impact on the field of sports (39). Anti-pandemic measures have affected students involved in sports, their sports life and training arrangements, leading to physical and psychological problems (40). Athletes have had to adapt to living in isolation, limiting their usual activities, uncertain prospects and anxiety about their training schedule (41). How do student athletes react, how do they live, how do they cope with the situation–such questions require answers based on objective data (42).

Comparative studies conducted during implementation of various anti-pandemic measures already provided some information (42). One of the examples includes Scotland and Japan, countries with different anti-pandemic strategies (more restrictive in Scotland). However, lifestyle restrictions have been found to affect adolescents' health behavior and well-being in both countries (43). Other examples are comparative studies of anxiety and stress among Russian and Belarusian university students (22), as well as Russian and Bulgarian students (44). Government approaches to anti-pandemic efforts differed in these countries. Authors concluded that COVID-19 quarantines led to increased levels of fear, developing negative psycho-emotional states and increased use of psychoactive substances (22).

We aimed to describe changes in physical activity and differences in mental health indicators according to the criteria of life satisfaction, anxiety levels, stress perception, and choice of coping strategies among respondents in relation to the different anti-pandemic strategies of two neighboring countries. This is to our best knowledge the first comparative study evaluating the physical activity level and mental health status of student athletes after implementing strict anti-pandemic measures policy (Poland) and minimal restrictions (Belarus). We were able to compare and evaluate the situation in geographically close regions by singling out a specific group for comparison—physical education and sports students from partner universities. We found some specificity in the analyzed indicators, depending on the level of anti-pandemic measures. Results referring to physical activity were to some extent worse than other studies among student athletes (45). One of them (44) including two samples of adolescents concluded that the physical and mental state of student athletes was significantly more favorable than that of students who did not participate in sports. Our results suggest that mild anti-pandemic measures in Belarus and more restrictive ones in Poland have had little effect on the dramatic decline in PA in student athletes. This lack of differences between the two groups may stem from the fact that students from Poland tended to optimize their physical activity in the face of restrictions while students from Belarus tended to limit their activities to reduce their risk of infection (46). This is consistent with previous studies showing unexpected positive results from restrictions (47, 48) such as adolescents paying more attention to health, sports and recreational activities during the restrictions (49). Another study on students from Bulgaria reported that over half of them (58%) were physically active with high and elevated PA according to the IPAQ. Only 16% of the respondents did not exercise at all (50).

Statistically significantly higher levels of PA were recorded in a group of student athletes from Belarus in terms of intense physical activity and walking. Moreover, in the female student group—also in terms of walking and moderate physical activity, with priority for female students from Belarus. Calculations of total physical activity confirm this. There were no differences in MET rates among men of the two groups. On the contrary, women in Poland had a 1.5-fold lower total MET level compared to female students from Belarus.

In terms of mental health indicators, however, the surveyed student athletes in both countries coped relatively well. Regardless of restrictions level, the respondents felt quite energetic, alert and motivated. Similar results during lockdowns were also observed in other studies as well (51). Our participants that reported higher PA during the COVID-19 pandemic also showed higher levels of life satisfaction. The maximum level of satisfaction with life was declared by almost half of the students (48.3%). On the three-level scale, a low level (SWLS < 19) was recorded in one-fifth of all observations (17.3%). Scores indicating an average score (SWLS = 20–25) were found to be typical for 27.8% of all respondents. Belarusian students, however, varied significantly in higher levels of life satisfaction. These differences characterized both men and women.

Most of those surveyed managed to avoid serious consequences for their mental health. However, for some students, this period was accompanied by negative psychological effects. Higher levels of anxiety as a trait were associated with higher levels of anxiety as a state. In our study, higher anxiety levels, both as a trait and a state, were found in women. This applied to both countries. These results are consistent with previous data from the literature (52, 53) which described women as tending to present higher anxiety levels. An association was also found between physical activity and anxiety, with low levels of physical activity leading to increased anxiety scores in the COVID-19 study. These results seem to indicate that women are more likely to experience higher levels of anxiety regardless of preventive measures being used, underscoring the importance of sex-specific decision-making.

Given the unknown COVID-19 situation we may face in the future, the population should be aware of this possible difference and increased anxiety during restrictions, especially among women (54). When evaluating the results according to physical activity in three categories recommended by the IPAQ questionnaire, we found that those who reported greater PA, had lower levels of anxiety (personality trait and state). These indicators seem to underscore the evidence in the literature for a potential role for PA (43).

Thus, our results support the idea that people who turn to health-promoting behaviors, such as practicing more PA, have also higher life satisfaction and lower levels of anxiety. We also confirmed association between sex and anxiety which is consistent with other studies (55). In our study, however, the association was equally strong in both groups and was not sex-specific.

We also found that maintaining optimal physical activity during COVID-19 prevents negative stressors. In both groups, participants used appropriate strategies at cognitive and behavioral levels to handle negative situations.

In April 2020, a cross-cultural survey was conducted on a sample of 310 athletes of various disciplines from different countries in Europe, Asia and America. The results indicated that levels of stress were relatively low in athletes while using active coping strategies contributed to reduction of negative emotional states (56). Based on post-traumatic stress theory and the resource approach, athletes with higher levels of personal resources and resilience perform better in difficult situations than those with higher levels of stress and lower levels of self-control (57). Being a part of the student athlete group from Poland is associated with infrequent use of such integral coping strategies as “Avoidance” and “Seeking support.” Nevertheless, the choice of these strategies is more frequent among Belarusian respondents. This may be due to the media's muted coverage of COVID-19 in the first months of the pandemic. At that time, students could not actively confront the growing number of the infected.

The results allowed us to identify trends in the use of coping strategies according to gender. Female students are more likely to use coping strategies related to emotions and focusing on negative experiences. They are also characterized by a desire for social support. They seek advice, help and information, which may be related to the uncertainty of the situation itself; they have higher levels of stress perception compared to male students, as documented in our earlier studies (58).

The resource-based approach is an important area for further research on the problem of enhancing young people's adaptation to the uncertainties of the modern world and increasing their psychological well-being. Studies confirming the greater adaptive capacity of young people who play sports and are involved in sports activities also show the importance of this factor in developing appropriate coping strategies when faced with uncertainty (59). Our results emphasize the importance of determining which groups may face more difficulties during the on-going COVID-19 pandemic, its future waves and other potential pandemics. Identifying these vulnerable groups will help better target intervention strategies. Further research is needed to contribute to developing and implementing public health interventions that would encourage different types of physical activity in different social groups (60).

### Limitations

The study has several limitations. The data came from online surveys that are prone to well-known limitations, however our target group was well defined and very narrow mitigating some the methodological risks. The online recruitment could also be subject to response bias as we were not able to determine what factors encouraged or discourage respondents from participation. Finally, the cross-sectional design of the study that cannot determine the causality between variables. Nevertheless our sample size was large and results were consistent with current research on mental health during pandemics. Future studies may employ not only self-assessment tool, but also more objective measures to monitor physical activity levels and mental health indicators.

## Conclusions

In line with our initial hypothesis, practicing sports and increased physical activity in students during the COVID-19 pandemic was associated with mechanisms that contribute to preventing affective disorders. Belarusian student athletes had a lower frequency of subclinical manifestations of anxiety disorders, higher levels of satisfaction with life and greater resilience to stress compared with their Polish counterparts. Regardless of the physical activity level, behavioral coping among participants was dominated by constructive coping strategies with a predominantly active approach to the problem. Belarusian respondents often denied the actual nature of stressfuls situation by ignoring it, which may be related to less stringent anti-pandemic policies.

## Recommendation

Comprehensive analysis of all the effects caused by the COVID-19 pandemic will the subject of studies for years to come. Knowledge on specific cognitive and behavioral coping strategies used by athletes combined with assessment of their effectiveness may greatly assist psychologists, coaches and athletes in their search for the most appropriate solutions to the psychological issues. The results may help design and target interventions to increase physical activity associated with pandemic mitigation efforts. The results provide a valuable reference point in the search for the most effective approaches to athletes' coping with the consequences of pandemics in the future. They may also be used by psychologists and coaches in analyzing and understanding the impact of various situations on athletes' behavior and providing appropriate support. Public health officials and health policy experts should take these differences into account when evaluating COVID-19 containment measures. Future studies should determine whether our findings are applicable to other groups of young people and whether health may be improved by providing opportunities for young people to engage in sports and physical activity during pandemic-related restriction.

## Data availability statement

The raw data supporting the conclusions of this article will be made available by the authors, without undue reservation.

## Ethics statement

The studies involving human participants were reviewed and approved by the Bioethics Committee of the Medical University of Bialystok (protocol code APK.002.193. 2022). All of the subjects gave their informed consent for inclusion before they participated in the study. The patients/participants provided their written informed consent to participate in this study.

## Author contributions

AS: conceptualization, methodology, formal analysis, investigation, data curation, visualization, and writing—original draft. DS: formal analysis and investigation. EK-K and MC: methodology, formal analysis, and investigation. BK: investigation, writing—review and editing. FK, SS, JO, and JK: investigation. KK: methodology, formal analysis, and writing—original draft. All authors assisted in the preparation of the manuscript. All authors have read and agreed to the end version of the manuscript.

## Funding

The research and this article were funded by a Grant of the Polish National Agency for Academic Exchange, BPN/SZN/2021/1/00004.

## Conflict of interest

The authors declare that the research was conducted in the absence of any commercial or financial relationships that could be construed as a potential conflict of interest.

## Publisher's note

All claims expressed in this article are solely those of the authors and do not necessarily represent those of their affiliated organizations, or those of the publisher, the editors and the reviewers. Any product that may be evaluated in this article, or claim that may be made by its manufacturer, is not guaranteed or endorsed by the publisher.
